# Predictors of Undiagnosed Diabetes in the Bangladeshi Female Population: A Propensity Score–Weighted Machine Learning Analysis of BDHS Biomarker Data

**DOI:** 10.1155/jdr/2162121

**Published:** 2026-06-22

**Authors:** Nahid Sultana, Kanis Fatama Ferdushi

**Affiliations:** ^1^ Department of Statistics, Shahjalal University of Science and Technology, Sylhet, Bangladesh, sust.edu; ^2^ Department of School of Medicine and Population Health, University of Sheffield, Sheffield, South Yorkshire, UK, sheffield.ac.uk

**Keywords:** 2022 BDHS, diabetes mellitus, explainable AI (XAI), nomogram, propensity score, public health

## Abstract

**Background:**

In Bangladesh, diabetes mellitus has become a substantial public health burden, imposing a strain on the population both economically and clinically. Robust methods for identifying high‐risk populations using national data are urgently needed due to the increasing prevalence of metabolic noncommunicable illnesses throughout South Asia. Using data from the most recent wave of a reliable national survey, this study was aimed at creating and testing a diabetes prediction model for adult female Bangladeshis.

**Methods:**

The 2022 Bangladesh Demographic and Health Survey (BDHS) biomarker data were examined, where key factors of undiagnosed diabetes were identified using stacked ensemble machine learning (ML) algorithms together with explainable AI (XAI) approaches after adjusting for selection bias using a propensity score model. A nomogram, a user‐friendly clinical tool for risk assessment, was created. The area under the receiver operating characteristic (AUROC) curve was used to assess the model′s performance.

**Results:**

Of the 18,547 weighted female participants (unweighted *n* = 7833), the prevalence of undiagnosed diabetes was 6.26% (95% CI: 5.52%–7.00%). Significant predictors identified included age (mean 42 ± 16 years vs. 38 ± 16 years; *p* < 0.001), BMI (mean 24.7 ± 4.6 kg/m^2^; *p* < 0.001), and hypertension (mean 120/78 mmHg; *p* = 0.001). ML models demonstrated high predictive accuracy (AUROC > 0.80), and a simplified clinical nomogram was developed to provide personalized risk scores. XAI (SHAP) analysis emphasized nonlinear influences, particularly in urban residents, who accounted for 40% of undiagnosed cases, and the richest quintile, which bore a disproportionate burden of 33% (*p* < 0.001) compared to just 13% in the poorest quintile.

**Conclusion:**

For early diabetes screening in Bangladesh, the established predictive model and nomogram provide an evidence‐based method. These technologies can help healthcare providers and policymakers tailor interventions toward high‐risk groups by using nationally representative data. This could potentially lessen the sustained health and financial effects of the diabetes epidemic.

## 1. Introduction

Bangladesh is experiencing a major shift in health profile, where diabetes stands as a major emergency. In 2024, almost 13.1 million cases were recorded, whereas by 2045, the number is expected to nearly double [1]. However, the most concerning part of this trend is how many people are being left behind by the healthcare system. According to the Bangladesh Demographic and Health Survey (BDHS), as many as six in 10 cases go undiagnosed, which implies that a huge portion of the population is unknowingly moving toward cardiovascular disease or vision loss without any medical intervention to stop it [2].

The undiagnosed diabetes is no longer just a medical oversight; it is a major economic drain due to the financial and physical toll it causes. If we measure the damage caused by diabetes in terms of “productivity‐adjusted life years” or PALYs, the projected hit to the GDP could reach a staggering $97 billion over the current generation′s working life [3]. Bangladesh spends $217 million annually on direct care for diabetes [4]; therefore, it is clear that early detection is the only viable escape from the “wealth‐to‐poverty” trap these chronic illnesses create.

Although the incidence of diabetes is rising, Akter et al. [5] identified that a staggering number of cases in Bangladesh remain undetected. There still remains a significant geographic and diagnostic gap in diabetes screening in Bangladesh. Urban areas like Dhaka benefit from more screening camps; on the other hand, the rural periphery continues to be a “diagnostic desert” and lacks adequate infrastructure and trained personnel, which is home to the majority of the population. Traditional diagnostic methods, such as glycated hemoglobin (HbA1c) and fasting blood glucose (FBG), are frequently out of reach for rural families due to cost and travel distance. To bridge this gap, we need to move toward noninvasive, low‐cost screening instruments. By utilizing anthropometric and demographic data, we can create tools that work where traditional laboratories cannot.

While researchers often rely on WHO‐recommended body mass index (BMI) thresholds and standard clinical markers, research indicates that these international criteria considerably underestimate metabolic risk in the unique Bangladeshi setting. Furthermore, as Khan et al. [6] point out, traditional logistic regression is not enough to map the messy, overlapping relationships between age, hypertension, and urbanization. We need more sophisticated predictive models that can actually function where they are needed most: in rural areas where clinical resources are scarce.

Earlier studies on automated diabetes screening in Bangladesh used several machine learning (ML) techniques, such as decision trees and support vector machines (SVMs) [7]; however, most of these studies focused mainly on clinical information, with limited attention to social, economic, and geographic factors that can influence undiagnosed diabetes, particularly in rural and changing communities. Additionally, the use of “complete case analysis,” which ignores the nonrandom nature of biomarker participation, remains a serious flaw in research employing national survey data such as the BDHS. Furthermore, the extreme class imbalance—where healthy respondents greatly outnumber undiagnosed diabetics—is not taken into consideration by many predictive models, resulting in low sensitivity. In particular, our study uses the synthetic minority oversampling technique (SMOTE)–driven balancing and inverse probability weighting (IPW) to close these gaps.

While predictive accuracy is improving through the use of ML models, a significant barrier remains regarding clinical trust. Healthcare providers often resist “black‐box” models because they do not reveal the “why” behind a diagnosis. The current study addresses this challenge by integrating SHapley Additive exPlanations (SHAP), a framework for explainable AI (XAI). SHAP allows for a move beyond broad statistics to examine feature importance on an individual level [8]. This transition from identifying “what” the risk is to explaining “why” it exists is currently missing from Bangladeshi diabetes research, leaving a critical gap between advanced modeling and practical clinical application.

Although Random Forest (RF) and Extreme Gradient Boosting (XGBoost) are powerful ML tools, their internal logic is often concealed as a black box, making them difficult for healthcare workers to trust. A nomogram addresses this by converting complex results into a clear, visual interface with a point‐based system. Iasonos et al. [9] argue that this approach is actually superior to traditional staging systems for calculating risk. Furthermore, in resource‐limited settings like rural Bangladesh, where electricity and internet are unreliable, the nomogram serves as a vital “paper‐based” tool that allows for high‐level predictive accuracy even in the absence of digital infrastructure [10–12]. Global health recommendations frequently include “one‐size‐fits‐all” cut‐offs (e.g., age > 40 or BMI > 25). On the other hand, South Asians have a constant gradient of diabetes risk. Nomograms enable a customized risk score (0%–100%) based on each patient′s unique combination of risk factors [13].

Even though earlier research has used BDHS data to examine the prevalence of diabetes in Bangladesh, significant gaps remain. First, current models often overlook nonrandom participation in biomarker collection, which can introduce selection bias; our study addresses this by applying IPW. Second, previous ML efforts have struggled with the extreme class imbalance typical in metabolic surveys, a challenge we counter using SMOTE. Finally, there is a shortage of translational tools that convert complex AI outputs into practical clinical formats; we bridge that gap by synthesizing ensemble‐learning insights into a user‐friendly nomogram for low‐cost screening.

The remainder of this paper is organized as follows: Section 2 reviews the related literature and defines our study objectives; Section 3 details the methodology, including bias correction, class balancing, the development of the stacked ensemble ML pipeline, and the creation of the clinical nomogram. Section 4 presents the results, Section 5 discusses the clinical implications of our findings, and Section 6 describes concluding remarks with study limitations in Section 7.

## 2. Literature Review

The Bangladeshi population is particularly vulnerable to the “South Asian phenotype,” which is marked by excessive visceral fat, low muscle mass, and increased insulin resistance even at lower BMI levels [2]. A wealth paradox has been revealed by Bangladeshi studies in which rising socioeconomic status is found to be positively connected with undiagnosed status due to sedentary lifestyle changes and dietary transitions [2, 14–16]. This stands in contrast to Western societies, where obesity is the main cause of diabetes [17].

On a global scale, ML algorithms like RF and SVMs are setting new standards for accuracy. When it comes to predicting diabetes, ML has already shown it can outperform traditional scoring systems. Similar applications have been observed in Bangladesh; for instance, Chowdhury et al. [18] used basic ML models to analyze the 2017–2018 BDHS data. Ishtiaq et al. [19] identified hypertension as the predictor for diabetes using ML methods using the 2022 BDHS dataset, while Mondol and Majumder [20] focused on the BDHS 2017–2018 dataset using neural networks (NNs) and grid search hyperparameter tuning. Additionally, Tasin et al. [7] specifically investigated diabetes among females using XGBoost combined with the ADASYN oversampling technique. Al‐Zoba et al. [12] explored automated diabetes prediction using a variety of ML classifiers. However, a critical flaw persists across these studies: the reliance on “complete case analysis,” which fails to account for systematic nonresponse bias.

Additionally, nonresponse in biomarker data is not merely a clinical gap but a significant source of selection bias, as the refusal to provide blood samples often correlates systematically with a participant′s social standing. While most studies rely on complete case analysis, Mondal et al. [21] showed that those who opt out frequently come from different socioeconomic strata than those who participate. This means those who are tested differ systematically from those who are not, leading to a “selection bias” that traditional linear models cannot adequately address.

While ML models are capable of addressing the selection bias, current ML models for Bangladesh employ complete case analysis, which overlooks this issue [8, 22]. Therefore, a significant methodological gap remains common across these studies: These models rely on a standard clinical sample and do not account for the complex survey participation biases inherent in the BDHS that can skew the results. Furthermore, the unique Bangladeshi phenotype for diabetes involves complex, nonlinear relationships that these traditional models fail to grasp. To solve this, the current study applies IPW to bridge the gap between traditional econometrics and modern ML [23].

Furthermore, although high‐performance algorithms like XGBoost and NNs have been explored [7, 20], they often function as “black boxes” with limited clinical utility. There is a notable absence of research that translates these complex nonlinear relationships into interpretable clinical tools. This study bridges these gaps by integrating IPW to correct for selection bias and deploying a SHAP‐based clinical nomogram to provide a practical, representative risk assessment tool for resource‐limited settings. Given the methodological gaps and the lack of translational screening tools identified in the preceding sections, the primary objective of this study is to develop a robust, bias‐corrected predictive framework. The primary contributions of this study are summarized as follows:

• This study uses IPW to reduce selection bias in blood test participation and provide a more accurate estimate of the national diabetes burden.
• A stacked ensemble model combining RF, XGBoost, and a logistic meta‐learner was developed to better capture complex relationships between socioeconomic factors and metabolic health.
• SMOTE was applied to address class imbalance and improve the model′s ability to identify undiagnosed diabetes cases.
• SHAP was used to make the ML model more interpretable and to identify important socioeconomic factors linked to diabetes, including the “wealth–diabetes paradox” in Bangladesh.
• Finally, the ML findings were translated into a simple, noninvasive clinical nomogram that can help community health workers identify high‐risk individuals in resource‐limited settings.

## 3. Methodology

### 3.1. Data Source and Study Population

The BDHS biomarker subsample, which included individuals 18 years of age and older, was used for this study. To make the analysis nationally representative, BDHS data were utilized, which follows a cross‐sectional two‐stage stratified cluster sampling survey design. The National Institute of Population Research and Training (NIPORT) in Bangladesh administered the 2022 BDHS, a nationally representative survey (NIPORT and ICF, 2024). It served as a reliable tool for studying health‐related problems since it systematically collected information on a broad spectrum of demographic, socioeconomic, and health indicators. Both the Bangladesh Medical Research Council (BMRC) and the ICF Institutional Review Board ethics committee had approved the protocol for the 2022 BDHS [2].

Initially, *7859* female participants were identified from the BDHS biomarker database. However, 26 cases were excluded due to “unknown” or missing values in the education covariate and FBG records. As these excluded cases represented a negligible fraction of the total sample (*< 0.4%*), their removal was unlikely to introduce significant selection bias or affect the national representativeness of the findings. This resulted in a final analytical unweighted sample of *7833* women and was used for the final analysis. Specifically, we analyzed the “Household Member” Recode dataset (PR file), which contained information on all de facto and de jure members of the surveyed households. The survey′s subsampling methodology was responsible for the exclusion of male participants, as the survey gave preference to female respondents for FBG and anthropometric data.

Undiagnosed diabetes, which was the response variable for this study, was defined as FBG level ≥ 7.0 mmol/L, which is equivalent to FBG ≥ 126 mg/dL (or HbA1c ≥ 6.5*%*) among individuals who reported no prior diagnosis by a healthcare professional, in accordance with the World Health Organization (2019) criteria and the methodology used in the most recent BDHS (NIPORT and ICF, 2024) [24].

### 3.2. Statistical Analysis

#### 3.2.1. Bias Correction: IPW

An IPW approach was used to mitigate potential selection bias resulting from nonparticipation in biomarker testing. First, a propensity score model was developed using logit regression. The probability (*P*) of a person agreeing to and finishing the blood glucose test was calculated using a propensity model based on demographic factors: age, residence, wealth, education, BMI, and division.

The following logit model was used to estimate the probability (*P*):

logitP=lnP1−P=β0+β1Age+β2Residence+β3Wealth+β4Education+β5BMI+β6Division

where *β*_0_ is the intercept and *β*_1_ … *β*_6_ are the regression coefficients for each predictor. The model uses a *quasibinomial* distribution to account for the overdispersion inherent in complex survey weights.

As normal logistic regression implies that all observations are independent and identically distributed, it is frequently incorrect in the setting of the BDHS. However, the data showed a “clustering effect” as a result of the multistage cluster sample design, where people in the same households and neighborhoods exhibited intracluster similarity, resulting in a variance that is substantially bigger than the mean—a phenomenon known as “overdispersion.” Our approach takes this into account by adding a dispersion parameter that “expands” the standard errors in a quasibinomial regression model with the logit link function. Inclusion of the quasibinomial distribution guaranteed that the *p* values and confidence intervals were more robust and conservative, appropriately representing the uncertainty brought about by the intricate survey weights and the nonindependent nature of the sampled population.

In the final step, the IPW method was used to determine the final analytical weights, that is, a “super weight,” in order to account for potential selection bias resulting from nonparticipation in the biomarker module. First, each respondent′s participation probability ($\overset{\wedge }{p}$) was calculated using the previously mentioned weighted logistic regression model, which was adjusted for physical and sociodemographic traits. Then the original BDHS sampling weight (*W*_sampling_) was multiplied by the inverse of the calculated probability of participation for those who finished the module ($W_{\text{final}}=W_{\text{sampling}}*1/\overset{\wedge }{p}$) to determine the final analysis weight (*W*_final_). The weight was set to zero for those who did not take part. The final weight was calculated as follows:

Wfinal=Wsampling×1Pparticipation, if participated=100, if participated=

#### 3.2.2. Class Balancing Using SMOTE

Within the BDHS dataset, the frequency of undiagnosed showed an inherent class imbalance, where healthy persons outweighted the minority positive class, that is, people with undiagnosed diabetes. In the training set, the minority class (undiagnosed diabetes) represented only 6.25% of the total samples. To mitigate this “lopsided” distribution, SMOTE was applied to the training data that created synthetic data by interpolating between existing minority cases and their *k*‐nearest neighbors (*K* = 5 nearest neighbors). The testing set remained unadjusted to ensure that the model′s performance metrics reflected its capability to identify cases within the true, imbalanced distribution of the Bangladeshi population. SMOTE was selected over simple oversampling, which merely duplicates existing observations and increases the danger of overfitting. This procedure broadened the decision boundary of the ML ensemble, ensuring that the model prioritized clinical sensitivity while avoiding a prediction bias toward the majority healthy class.

To maintain the representative nature of the BDHS data within the ML framework, original sampling weights were reintegrated into the training task using a weight‐aware classification learner in the mlr3 R package. In order to ensure that the predictive model took into consideration both data imbalance and complex survey design, synthetic samples were given a unit weight while original observations retained their calibrated analytical weights.

#### 3.2.3. The ML Pipeline: Stacked Ensemble Method

Following data augmentation with SMOTE, a stacked ensemble architecture was built with the R package mlr3 pipeline framework. Stacking combines the strengths of different algorithms, reduces the weaknesses of individual models, and helps the system make more accurate and reliable predictions. To maximize predictive performance, we employed a stacked generalization (blending) architecture where two high‐performing base learners were trained as Level‐0 experts. Their cross‐validated probabilities were then integrated by a Level‐1 logistic meta‐learner to reduce the variance inherent in single‐model approaches as well as to provide the probabilistic framework necessary for threshold optimization via Youden′s Index.

1. Level‐0 (base learners): Two distinct ML algorithms were chosen to capture complex patterns in the survey data: RF (via the Ranger package), which excels at dealing with high‐dimensional noise and nonlinear relationships, and XGBoost, which is optimized for identifying complex interactions between clinical and sociodemographic features. To prevent data leakage and overfitting, the base learners were wrapped in a fivefold cross‐validation (CV) layer that computed out‐of‐sample class probabilities for the full training set.
2. Level‐1 (meta‐learner): At this stage, the probabilities generated at Level‐0 were combined with a logistic regression meta‐learner. This Level‐1 model added optimal weights to the base learners′ predictions to reduce log‐loss and produce a single, robust probability score for undiagnosed diabetes.
3. Survey‐weighted training and optimization: At this stage, the final analysis weights (which include both BDHS sampling weights and IPW participation weights) were fed directly into the training task. This guaranteed that the ensemble′s decision bounds reflected the national population. Finally, rather than employing the traditional 0.5 classification threshold, this study used the “Youden Index” to find the best probability cutoff that balances clinical sensitivity and specificity.

#### 3.2.4. Model Interpretability (XAI)

The stacked ensemble functioned as a model with constrained transparency. In order to transform it from a complex model to a clinically interpretable instrument, the study used SHAP. SHAP applied coalitional game theory to break down the model′s output into the total of the impacts of each individual attribute. In comparison to typical global relevance metrics (e.g., Gini impurity or gain), which merely provide an aggregate ranking, SHAP offered several specific advantages for clinical research: SHAP used directionality, which indicates whether a feature (e.g., increasing BMI) increased or decreased the likelihood of undetected diabetes. In addition to this, SHAP used consistency, which assures that characteristics with the greatest impact on the model were always given a greater priority rating, independent of the model′s underlying design. Furthermore, SHAP provided both local and global explanations. While this study used global feature rankings to identify the fundamental drivers of undiagnosed diabetes in the Bangladesh population, SHAP also preserved “local faithfulness,” which allowed for the explanation of specific high‐risk instances.

#### 3.2.5. Development of the Clinical Nomogram

The high‐dimensional ML model was reduced to a clinical nomogram for practical use in Bangladeshi settings with limited resources. This study developed a noninvasive method that enables practitioners to determine a patient′s likelihood of having undetected diabetes using only physical measurements and demographic information by mapping the log‐odds of the ensemble′s output onto a point‐based visual scale [25]. The nomogram was constructed by converting the log‐odds of the model′s coefficients into an understandable scoring system. Each clinical and sociodemographic variable, such as systolic blood pressure, BMI, and age, was given a weighted point value. By adding these points, healthcare workers can create a “total point” score, which directly correlates to the likelihood of undetected diabetes. The nomogram is intended for use by community health workers and practitioners in rural or underserved areas of Bangladesh, where FBG testing is often limited.

A multivariable logistic regression model for the nomogram where the most significant variables revealed by the stacked ensemble were included. While the ensemble produced the highest predicted accuracy, the logistic model was chosen as the final tool to provide transparency and enable the transfer of model coefficients into a point‐based visual scale.

The multivariable logistic model used to build the nomogram also incorporated the super weights (sampling weights × IPW) to ensure the nomogram was not biased toward any specific subgroup. In the nomogram, the points for each variable were derived from the model′s coefficients (*β* values). The total points were mapped to a sigmoid function (the logistic function) to calculate the final probability. The logistic model used for the nomogram is as follows:

lnP1−P=β0+β1Age+β2Residence+β3Wealth+β4Education+β5BMI+β6Division+β7Systolic_bp+β8Diastolic_bp

**Figure 1:**
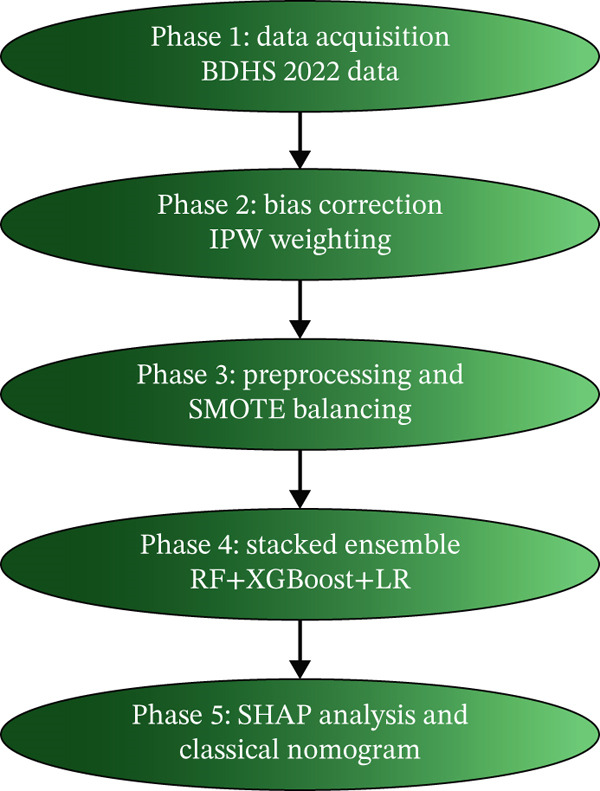
Operational framework of the study.

The following flowchart (Figure 1) depicts the integrated approach beginning with data acquisition and bias correction via IPW, followed by SMOTE‐based class balancing, the construction of a two‐level stacked ensemble (blending) model, and the final translation of AI insights into a clinical screening nomogram.

#### 3.2.6. Software

Statistical analyses were conducted in R (Version 4.3.1).

## 4. Results

Table 1 presents the baseline demographic and clinical characteristics of the study′s female population, stratified by diabetes status.

**Table 1:** Baseline demographic and clinical characteristics of the study population, stratified by diabetes status.

Characteristic	Overall (*N* = 18,547)^a^	Healthy/known (*N* = 17,387)^a^	Undiagnosed diabetic (*N* = 1160)^a^	*p* value^b^
Age	38 (±16)	38 (±16)	42 (±16)	< 0.001
Residence				< 0.001
Urban	2688 (27%)	2464 (26%)	224 (40%)	
Rural	5145 (73%)	4872 (74%)	273 (60%)	
Wealth				< 0.001
Poorest	1387 (18%)	1323 (19%)	64 (13%)	
Poorer	1478 (20%)	1408 (21%)	70 (14%)	
Middle	1516 (20%)	1426 (20%)	90 (19%)	
Richer	1634 (21%)	1527 (20%)	107 (22%)	
Richest	1818 (21%)	1652 (20%)	166 (33%)	
Education				0.12
No education	2080 (24%)	1927 (24%)	153 (28%)	
Primary	1882 (23%)	1761 (23%)	121 (24%)	
Secondary	2765 (38%)	2614 (39%)	151 (33%)	
Higher	1103 (15%)	1031 (15%)	72 (16%)	
Unknown	26	26	0	
BMI	23.0 (±4.3)	22.9 (±4.2)	24.7 (±4.6)	< 0.001
Systolic_bp	117 (±19)	116 (±19)	120 (±21)	0.001
Unknown	64	47	18	
Diastolic_bp	77 (±10)	77 (±10)	78 (±11)	0.039
Unknown	64	47	18	
Division				< 0.001
Barishal	854 (6.5%)	800 (6.5%)	54 (6.3%)	
Chattogram	1131 (18%)	1059 (18%)	72 (16%)	
Dhaka	1075 (23%)	959 (22%)	116 (37%)	
Khulna	988 (12%)	927 (12%)	61 (10%)	
Mymensingh	886 (8.4%)	861 (8.7%)	25 (4.0%)	
Rajshahi	990 (13%)	928 (13%)	62 (10%)	
Rangpur	940 (12%)	888 (12%)	52 (10%)	
Sylhet	969 (7.0%)	914 (7.1%)	55 (6.3%)	

^a^Mean (±SD). Total *N* reflects weighted frequencies to maintain national representativeness (percentage).

^b^Design‐based Kruskal–Wallis test; Pearson′s *X* ^2^: Rao and Scott adjustment.

### 4.1. Sample Characteristics and Prevalence

Of the 18,547 (weighted estimate; unweighted *n* = 7833) females examined, 1161 satisfied the criteria for undiagnosed diabetes with FBG ≥ 126 mg/dL with no prior diagnosis, which raises a serious public health issue. The undiagnosed group′s metabolic profile clearly differed from that of the healthy/known population, according to the weighted prevalence study.

### 4.2. Clinical and Anthropometric Divergence

Compared to their counterparts (38 ± 16 years; *p* < 0.001), the undiagnosed diabetic cohort showed a substantially different age profile, where the undiagnosed group was found to be comparatively older (mean 42 ± 16 years). Additionally, the undiagnosed group showed a higher mean BMI (24.7 ± 4.6 kg/m^2^) than the healthy group (22.9 ± 4.2 kg/m^2^; *p* < 0.001) according to anthropometric measures. Furthermore, the undiagnosed group was found to have significantly higher systolic and diastolic blood pressure (120/78 vs. 116/77 mmHg), both of which are clinical indicators of cardiovascular health, indicating a clustering of metabolic risk factors.

### 4.3. The Urban‐Wealth Intersection

According to a sociodemographic study, undiagnosed diabetes in Bangladesh was found to be disproportionately concentrated among urban and wealthy groups; despite making up a smaller percentage of the total sample, urban residents accounted for 40% of the undiagnosed cases.

The wealth gradient was especially noticeable, with prevalence rising steadily across wealth quintiles. The undiagnosed group contained 33% of all instances (*p* < 0.001) for the “richest” quintile, but only 13% of undiagnosed cases were found in the “poorest” quintile. This “wealth–diabetes paradox” suggests that sedentary behavior and dietary modifications such as increased consumption of processed foods, sugary drinks, and high‐calorie restaurant meals are causing this issue. Surpassing diabetes knowledge and screening may be linked to a greater economic status in Bangladesh.

Although place of residence and wealth quintile were found to be associated with undiagnosed diabetes cases, interestingly, formal education did not show up as a protective or risk‐defining factor. In the current Bangladeshi context, the risk of undiagnosed diabetes crosses educational barriers, as seen by the distribution of undiagnosed patients remaining largely constant across educational tiers (*p* = 0.12).

### 4.4. The Geographical Disparity

The cases of undiagnosed diabetes were found to exhibit marked regional heterogeneity across Bangladesh′s eight divisions (*p* < 0.001). Among all the divisions, the Dhaka Division emerged as the main hotspot, accounting for over one‐third of all undiagnosed cases (37%), in contrast to the Mymensingh Division, which showed the lowest relative burden, contributing only 4.0% to the undiagnosed diabetic cohort. There was a significant spatial disparity in the hidden burden of hyperglycemia, as evidenced by the roughly 3.4‐fold variation in undiagnosed diabetes cases between Dhaka and Mymensingh. Sylhet and Barishal divisions showed the second‐lowest undiagnosed prevalence estimates (6.3%), where other divisions, including Chattogram (16%), Khulna (10%), Rajshahi (10%), and Rangpur (10%), displayed a relatively proportional or slightly lower distribution of undiagnosed cases compared to their respective shares in the overall population.

Baseline comparisons were carried out utilizing design‐based tests, such as the Rao–Scott chi‐square for categorical variables, to take into consideration the intricate survey design of the BDHS. Unless otherwise noted, all *N* numbers and percentages mentioned relate to weighted frequencies because all analyses were conducted using survey weights to account for the complicated sampling design.

### 4.5. Undiagnosed Diabetes Prevalence

The weighted prevalence of undiagnosed diabetes in the adult female population of Bangladesh was 6.26% (95% CI: 5.52%–7.00%), which implied that a significant fraction of the diabetes population was not identified by the primary healthcare system.

The prevalence of undiagnostic diabetes was also assessed across all socioeconomic classes, where the burden of undiagnosed diabetes showed a clear rising trend (Table 2). The “poorest” and “poorer” quintiles had the lowest and same prevalence rates, at 4.24% (95% CI: 2.93%–5.54% and 3.18%–5.30%, respectively). The prevalence did, however, gradually rise through the “middle” (5.90%) and “richer” (6.58%) quintiles, where the “richest” quintile demonstrated a disproportionately higher burden, with a prevalence of 10.08% (95% CI: 8.11%–12.05%), which was more than double the rate observed in the poorest 40% of the population.

**Table 2:** Weighted prevalence of undiagnosed diabetes by wealth.

Wealth quintile	Prevalence (95% CI)
Poorest	4.24% (2.93%–5.54%)
Poorer	4.24% (3.18%–5.3%)
Middle	5.9% (4.53%–7.26%)
Richer	6.58% (4.87%–8.29%)
Richest	10.08% (8.11%–12.05%)

The place of residence was also found to be associated with undiagnosed diabetes prevalence. Compared to people living in rural regions, urban inhabitants had a significantly higher prevalence of undiagnosed diabetes (Table 3). In urban areas, the weighted prevalence was 9.22% (95% CI: 7.47%–10.97%), but in rural areas, it was significantly lower at 5.15% (95% CI: 4.36%–5.93%). This urban–rural disparity was found to be statistically significant, as evidenced by the nonoverlapping 95% confidence intervals. The result suggested that urban living is a key environmental factor contributing to Bangladesh′s undiagnosed diabetes burden.

**Table 3:** Weighted prevalence of undiagnosed diabetes by residence.

Residence	Prevalence (95% CI)
Urban	9.22% (7.47%–10.97%)
Rural	5.15% (4.36%–5.93%)

### 4.6. Propensity Score Model

The study used a weighted logistic regression model (quasibinomial family with a logit link function) to mitigate any selection bias and guarantee the representativeness of the female biomarker subsample. The binary dependent variable “participation in the biomarker module” was coded as 1 and nonparticipation as 0. To adjust the model for the confounding effect, important and available sociodemographic and physical variables, such as age, place of residence, wealth quintile, level of education, BMI, and division, were taken into account. BDHS sampling weights were applied to the model to adjust for the multistage cluster sampling strategy. To preserve the original data format for weight calculation, nonresponse and missing data were handled using the na.exclude function.

The odds ratio (OR) from the propensity score model is given in Table 4. From the table, it can be seen that education had a high impact on the probability of participation in the blood test. As the OR was less than 1, it means for every unit increase in the education level, the probability of participation in the blood test decreased by 21% (95% CI: 76%–82%). This suggested that using only the complete cases will make the analysis highly biased, as people with more education are more likely to reject the biomarker. It is also worth noting that the OR for wealth, residence, and BMI was greater than 1, which implied that people with higher wealth, residing in urban areas, and higher BMI had a higher probability of participating. Age and division did not show any contribution in the propensity score as the OR was almost exactly 1.0, and the confidence interval (0.99–1.001) crossed 1.0.

**Table 4:** Estimates from the propensity score model.

Estimates	(Intercept)	Age	Residence	Wealth	Education	BMI	Division
OR	0.27	0.99	1.08	1.06	0.79	1.04	0.99
2.5%	0.21	0.99	1.01	1.04	0.76	1.03	0.98
97.5%	0.35	1.001	1.17	1.09	0.82	1.05	1.01

**Figure 2:**
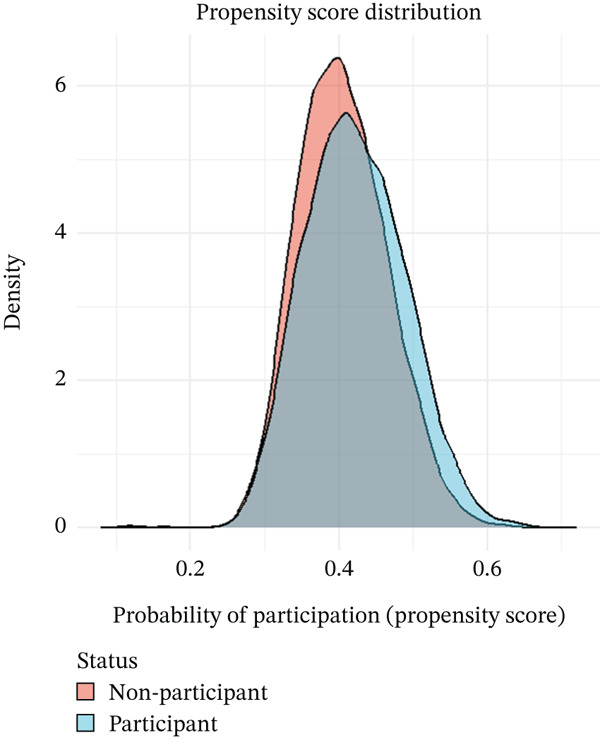
Probability of participation (propensity score).

To validate the robustness of the IPW, the common support region was evaluated through a density plot of predicted propensity scores (Figure 2). As depicted in Figure 2, a substantial distributional overlap was seen between the two groups, participants and nonparticipants, which implied that both groups were similar with respect to covariates after applying the propensity score. The nonoverlapping tails ensured that extreme variance was not seen during the calculation of weights.

### 4.7. SMOTE With a Stacked Ensemble Model

Table 5 shows how the SMOTE analysis balanced both groups before a stacked ensemble model was implemented. Before applying SMOTE, the data consisted of 93.74% of healthy/diagnosed people, where the undiagnosed group was a minority (only 6.25%). Such an imbalance in data could cause the risk of “majority class bias” with a stacked ensemble model. After applying SMOTE, the class distribution was approximately equal, which ensures adequate representation of the undiagnosed class. This equal distribution of the two classes allowed the stacked ensemble model to effectively estimate the risk patterns of the undiagnosed diabetic population.

**Table 5:** Class distribution of the response variable before and after applying SMOTE.

Class	Original count (imbalanced)	SMOTE count (balanced)
Healthy/known case of diabetes	5860 (93.74%)	5860 (51.70%)
Undiagnosed diabetes	390 (6.25%)	5474 (48.29%)

After applying SMOTE, the predictive performance of the stacked ensemble model was assessed as a population‐level screening tool. Predictive performance was evaluated using both conventional and optimized classification thresholds and is shown in Table 6. With an area under the curve (AUC) of 0.56, the model first showed a modest discriminating power with a sensitivity of 38% and a specificity of 68% using a conventional threshold.

**Table 6:** Predictive performance of the stacked ensemble model for undiagnosed diabetes under initial and Youden‐optimized classification thresholds.

Metric	Initial threshold	Youden‐optimized
AUC	0.56%	0.80%
Sensitivity	0.38%	90.1%
Specificity	0.68%	0.25%

The Youden Index was used to optimize the classification threshold in order to improve the model′s efficacy for community‐based screening when missing a potential case (false negative) entails a substantial clinical cost. Predictive priorities drastically changed as a result of this optimization. Nine out of 10 people in the population with undiagnosed diabetes were correctly identified by the Youden‐optimized model, which attained a high sensitivity of 90.1%.

A calculated trade‐off in specificity resulted from this increase in sensitivity, with specificity dropping to 25%. This performance profile described an efficient “wide‐net” screening tool in a public health setting: The model accurately identified the great majority of the at‐risk population for additional clinical testing while still excluding about one‐fourth of healthy individuals using noninvasive, economical predictors.

### 4.8. Global Model Interpretability (SHAP Analysis)

**Figure 3:**
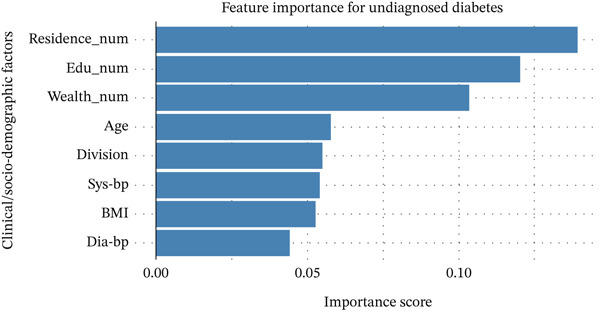
SHAP‐based feature importance ranking for undiagnosed diabetes.

SHAP values were computed to rank feature importance to facilitate the stacked ensemble′s decision‐making process. In order to identify cases of undiagnosed individuals, the model mostly relied on nonclinical, sociodemographic variables, as shown in Figure 3. In particular, the top 3 predictors of undiagnosed status were found to be type of residence, wealth index, and education level. Clinical indicators like BMI, age, and systolic blood pressure were also found to be important drivers despite the fact that their relative relevance scores were lower than those of socioeconomic and residential characteristics. This implied that in Bangladesh, an individual′s social status and surroundings were more reliable markers of undiagnosed diabetes than isolated clinical data. Although the inclusion of age and systolic blood pressure was consistent with accepted clinical knowledge, the higher ranking of wealth and place of residence highlighted the distinct socioeconomic context of diabetes prevalence in Bangladesh.

### 4.9. Clinical Application (Nomogram Development)

**Figure 4:**
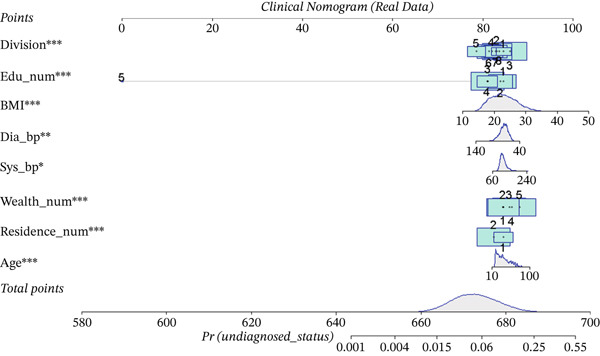
Clinical nomogram for predicting the probability of undiagnosed diabetes. ***p < 0.001, **p < 0.01, and *p < 0.05.

Based on the real‐world BDHS data, a clinical nomogram was developed, which will help to mitigate the disparity between computational complexity and clinical utility (Figure 4).

Based on statistical significance (*p* < 0.001 for the majority of variables, denoted by ∗∗∗), the nomogram allocated points to each variable, which in turn makes the complex model output into a straightforward point‐based system with the help of the visualization. A predicted probability (*P*) of having undetected diabetes, ranging from < 0.001 to 0.5, was closely correlated with each individual′s “total points,” which were calculated from their particular education level, BMI, blood pressure (both systolic and diastolic), wealth, residence, division, and age.

Based on the statistical significance, BMI, age, residence, education, division, and wealth emerged as the most influential predictors of undiagnosed status. Division and education level were the geographic and demographic elements that exerted the biggest impact on the risk score, with certain categories (such as Dhaka Division) adding the most points to the overall score, whereas significant nonlinear relationships were seen by biological markers, such as BMI and blood pressure (both systolic and diastolic), where greater values correlated with an increasing point tally. Factors like wealth, division, and education displayed a wide point margin, meaning they markedly changed the distribution of risk. While BMI and blood pressure were significant, their point distributions were relatively narrow, suggesting they acted as determinants that modulated the broader socioeconomic risk profiles.

By adding up the points given to each predictor′s value, the nomogram′s usefulness is illustrated in Figure 4. An individual with 680 total points, for instance, would map to a predicted probability (*P*) of roughly 0.25 (25%), reflecting a combination of a particular division, advanced age, urban domicile, and high BMI. The cumulative score was converted into a predicted likelihood of undiagnosed diabetes using the “total point” axis, which ranged from roughly 580 to 700. This system made it possible to quickly identify high‐risk people who ought to be given priority for FBG or formal HbA1c testing.

## 5. Discussion

This study was aimed at obtaining bias‐corrected prevalence estimates of undiagnosed diabetes among the female population in Bangladesh by employing IPW, subsequently training a SMOTE‐balanced stacked ensemble ML model whose complex logic was revealed through SHAP‐based feature importance and a clinical nomogram to provide a high‐sensitivity, interpretable screening tool for high‐risk cohorts. The findings from this study demonstrated the utmost importance of mitigating selection bias to ensure applicability to a broader female population of diabetes prevalence estimates across Bangladesh. Although BDHS data offer standard sampling weights for generalization in the broader population, these weights were scant for analyzing the biomarker subset, as participation in the blood glucose test was not missing completely at random (MCAR). This study overcame this by implementing IPW, which is developed to accommodate MAR.

By applying the reciprocal of the participation probabilities generated from a propensity score model, this study competently upweighted the group of people who were found to be statistically less likely to provide blood samples. This analytical adjustment ensured that the final predictive statistics captured the underlying population diversity to prevent bias against specific socioeconomic groups and provided a more precise basis for the subsequent ML analysis.

The weighted prevalence of 6.26% (95% CI: 5.52%–7.00%) for undiagnosed diabetes among female adults in Bangladesh revealed a critical diagnostic gap in the national healthcare infrastructure. This “silent” burden implies that basic healthcare screening procedures may not be adequate to keep up with the evolving epidemiological profile of the population. The lower bound of the confidence interval (5.52%) indicates that even in the most optimistic scenario, at least 5.5% of the population is living with untreated hyperglycemia, which significantly increases the national risk for long‐term complications such as retinopathy, neuropathy, and cardiovascular disease.

The prevalence of 6.26% of undiagnosed diabetes is significantly higher than regional data; for example, prior research in South Asia has frequently found lower undiagnosed rates; however, current trends in the 2017–2018 BDHS indicated an increasing trajectory in metabolic diseases [15, 26, 27]. The increase in undiagnosed diabetes prevalence can likely be attributed to swift urban expansion and food consumption pattern shifts. The critical need for thorough screening is highlighted by the fact that more than 1100 of the 18,547 (weighted estimate; unweighted *n* = 7833) female population satisfied the criteria for undiagnosed diabetes (FBG ≥ 126 mg/dL).

Our findings showed a clear wealth–diabetes paradox in Bangladesh, reflecting changes observed in other fast‐developing countries such as Vietnam and India [28, 29]. Economic transition is currently a risk factor for metabolic disease, as evidenced by the prevalence of 10.08% in the “richest” quintile and only 4.24% in the “poorest” quintile. This is consistent with international data indicating that diabetes is initially a “disease of affluence” in low‐ and middle‐income countries (LMICs) before moving toward the poor as the pandemic progresses [30].

Forty percent of all undiagnosed cases were concentrated in urban regions, where the frequency was 9.22% as opposed to 5.15% in rural areas. This result is in line with research conducted in Southeast Asia and sub‐Saharan Africa that links urbanization to the “nutrition transition”—a move toward processed foods and sedentary labor [31]. Clinical indicators clearly diverged in the undiagnosed group, which is indicative of “metabolic syndrome” clustering. The undiagnosed cohort was found to have a higher mean BMI (24.7 kg/m^2^ vs. 22.9 kg/m^2^) and was markedly older (mean 42 years vs. 38 years). Furthermore, the undiagnosed diabetes group′s clustering of higher systolic and diastolic blood pressures (120/78 vs. 116/77 mmHg) indicated increased cardiovascular risk even prior to a diabetes diagnosis. This intersection of comorbidities aligns with a prominent theme in recent epidemiological studies conducted throughout South Asia regarding the “double burden” of diabetes and hypertension [32].

With the aim of a comprehensive analysis of undiagnosed diabetes to overcome the limitations of standard statistical modeling, this study implemented SMOTE followed by a stacked ensemble model. SMOTE successfully addressed the pre‐existing class imbalance within the BDHS dataset, where the undiagnosed diabetes group was a small minority. The application of SMOTE ensured that the ML model could recognize individuals at elevated risk with equal sensitivity to the majority healthy population. A stacked ensemble model was then used on SMOTE‐adjusted reweighted training data to reduce bias and improve prediction accuracy by integrating learning from various approaches. The stacked ensemble ML model was able to reveal intricate, nonlinear links between anthropometric and socioeconomic factors. Finally, the study utilized SHAP to explain the model′s internal structure and evaluate the precise influence of each feature on a person′s risk profile for the purpose of progressing from a “noninterpretable model” prediction to meaningful clinical observation.

A classification threshold of 0.05 was used in the ensemble model evaluation to prioritize the model′s clinical utility as a primary screening tool. A standard default threshold of 0.50 would have produced an excessive misclassification due to false negatives, thereby missing a substantial proportion of high‐risk individuals, considering the relatively low occurrence of undiagnosed diabetes in the BDHS sample. The sensitivity of the model was increased by decreasing the threshold to 0.05, ensuring comprehensive coverage of potential cases during screening.

This approach was consistent with risk‐reduction priorities in public health since the costs incurred from follow‐up confirmatory testing were minor relative to the costs of failing to identify true cases. The initial threshold had allowed for a specificity of almost 68%, showing that the model correctly classified the vast majority of disease‐free individuals even with a liberal cutoff, which in turn mitigates diagnostic workload for medical staff. Subsequently, the Youden Index was employed as a standard benchmark for academic comparison, which used a stability‐focused classification by offering a balance optimization according to statistical criteria between sensitivity and specificity.

This study used SHAP for feature importance from the stacked ensemble ML model. The SHAP analysis highlighted a significant change regarding strategies for screening undiagnosed diabetes in Bangladesh. Although screening has traditionally been guided largely by clinical markers, our ensemble model predominantly relied on sociodemographic variables to detect previously undiagnosed cases. The ML model primarily leveraged residence, wealth, and education. This implied that environmental conditions and social position served as earlier warning indicators of disease risk than isolated clinical data within the Bangladeshi context. The leading impact of socioeconomic factors demonstrated a distinct change in epidemiological patterns in the area, even though the importance of age and systolic blood pressure remained in accordance with global clinical literature. These findings highlighted that affluent urban clusters should be the main priority of public health interventions. As lifestyle changes may occur more quickly in affluent urban areas than in clinical settings, this escalates the burden of undiscovered illness.

According to our findings, the major determinants of undiagnosed diabetes in the Bangladeshi cohort were the “social determinants of health” (SDoH). The nomogram showed that the majority of the risk distribution was driven by the broader socioeconomic context, notably wealth, education, division, and urban location, even though biological variables like blood pressure and BMI remained crucial for fine‐tuning risk. This implied that the socioenvironmental factors influenced how biological risk manifested within the Bangladeshi population. In this context, wealth, division, and education probably served as persistent behavioral modifications that were more informative for identifying undiagnosed diabetes than a single BMI reading at a clinic, such as high‐calorie diets and physical inactivity.

A more sophisticated and proactive public health response is made possible by this change from a strictly clinical to a socioclinical screening strategy, which enables doctors to find “hidden” cases based on easily accessible demographic profiles before serious metabolic issues develop. The nomogram revealed that in the BDHS cohort, “macroenvironment” (wealth, division, and place of residence) was a stronger predictor of undiagnosed status than “microbiological” markers (BMI and blood pressure). This emphasized how important a socioclinically integrated screening strategy is, and this finding underscored that in the Bangladeshi demographic, the “wealth paradox” acts as a primary driver of undiagnosed status, surpassing the predictive influence of individual hemodynamic measurements.

### 5.1. Comparison With Previous Studies

A critical comparison of our study with existing literature reveals several methodological advancements. Unlike Tasin et al. [7] and Al‐Zoba et al. [12], who treated the BDHS dataset as a static clinical sample, our study implemented IPW to correct for nonrandom biomarker participation bias; in addition to that, while previous models achieved high performance using single‐algorithm approaches like XGBoost, our stacked ensemble leveraged the complementary strengths of RF and XGBoost, blended via a logistic meta‐learner for superior generalization. Furthermore, while earlier studies focused primarily on model performance metrics, we translated these complex log‐odds into a visual clinical nomogram to mitigate the gap between ML theory and frontline screening in Bangladesh.

## 6. Conclusion

This study appropriately addressed the parallel issues of selection bias and model understandability in assessing undiagnosed diabetes among the female population in Bangladesh. After adjusting for nonrandom selection into biomarker testing using IPW, a substantial “silent” prevalence of 6.26% was revealed. Even under the most optimistic estimates, 5.52% of the population remains untreated for hyperglycemia, demonstrating a considerable diagnostic shortfall in the healthcare system.

Results from the study supported the wealth–diabetes paradox, which shows evidence of a notable association between undiagnosed status and urban residence (9.22%) and higher socioeconomic level (10.08% in the richest quintile vs. 4.24% in the poorest). These results indicate that diabetes is currently a “wealth‐associated health condition” in Bangladesh amid rapid urbanization and changes in dietary patterns. Moreover, this study addressed the limitations of conventional statistical methods to capture higher order, nonlinear effects between socioeconomic and biomedical factors by using a SMOTE‐balanced stacked ensemble ML model. This study provided a pragmatic and interpretable screening technique by emphasizing high sensitivity using a 0.05 threshold and offering a clinical nomogram. By reconciling differences between state‐of‐the‐art ML and real‐world clinical utility, our analysis will assist healthcare professionals in identifying and ranking high‐risk groups for life‐saving interventions.

## 7. Limitations of the Study

Although this study offers a strong framework for diabetes screening, it is important to acknowledge some limitations also. Firstly, due to the unavailability of the HbA1c biomarker in the BDHS data, FBG readings ≥ 126 mg/dL were used to diagnose undetected diabetes. Unlike HbA1c, which is standardized for long‐term monitoring, FBG values can be influenced by daily glucose fluctuations. Furthermore, the IPW adjustment′s efficacy depends on whether the variables in the propensity score model are adequate to capture the nonparticipation (missing at random). While the model is highly applicable to the female population in Bangladesh, the specific “points” and weightings in the nomogram may require recalibration before being applied to different regional contexts in South Asia. Due to data availability constraints, the nomogram could not be generated for the male population. According to the SHAP‐based feature significance, socioeconomic variables were found to be more effective at prediction than using clinical data alone, such as BMI. This could cause “false alarms” among wealthy people who lead healthy lifestyles; therefore, physicians should use the technique in conjunction with clinical judgment rather than in place of it.

## Author Contributions

N.S.: conceptualization, data curation, methodology, formal analysis, investigation, validation, visualization, writing—original draft, and writing—review and editing. K.F.F.: writing—original draft.

## Funding

No particular grant from any funding agency, business, or nonprofit organization was obtained for this study.

## Ethics Statement

The BDHS is carried out in accordance with ethical norms and guidelines and is frequently approved by both international and local ethical review boards in Bangladesh (such as the Ministry of Health and Family Welfare or the National Ethics Committee). This guarantees that the survey complies with internationally accepted ethical norms. A thorough explanation of the survey′s objectives, protocols, and data collection techniques is given to each participant. Researchers who utilize BDHS data commit to utilizing it only for statistical and research reasons, refraining from any attempts to reidentify specific persons.

## Conflicts of Interest

The authors declare no conflicts of interest.

## Data Availability

The data used in this study are third‐party data provided by the DHS Program. The raw biomarker and survey data are available to registered users at https://dhsprogram.com.
